# Consequences of COVid-19 in Latin American dentists in the first year of the pandemic, the period prior to vaccination campaigns

**DOI:** 10.1016/j.heliyon.2024.e24223

**Published:** 2024-01-11

**Authors:** Juan Gabriel Costa, Ana Beatriz Gaudio, Nicolás Gomez Giorgi, Camila Hanow

**Affiliations:** Cátedra de Microbiología, Facultad de Odontología, Instituto Universitario Italiano de Rosario (IUNIR), Argentina

**Keywords:** COVid-19, Latin America, Dentist, Pandemic, Survey

## Abstract

**Objectives:**

The aim of this investigation was to assess the impact of the COVID-19 pandemic on dentists in Latin America during the initial year of the outbreak, specifically within the timeframe preceding vaccination campaigns. This study determined the various facets in which dentists were affected and exactly what proportion of them was harmed.

**Methods:**

A comprehensive 33 question survey was distributed across 19 Latin American countries after the first year of the COVID-19 pandemic's presence in the region.

**Results:**

There was an absence of statistically significant differences in responses among the surveyed countries in Latin America, with the exception of four questions out of the total 33. Some relevant findings of Latin American were: one in three dental professionals experienced discrimination based on their occupation. Concurrently, three out of four dentists reported initiating new activities to lessen discomfort. Notably, 8.63 % of respondents sought assistance from a psychologist or psychiatrist, while 17.71 % resorted to the consumption of psychoactive substances within the first year of the pandemic.

Furthermore, 7.28 % of the professionals indicated that they still had not obtained all the necessary personal protective equipment for their work and 92.05 % disclosing that they personally financed these essential resources. A certain percentage of dentists stated that the quality of care decreased due to the implementation of the new safety measures (40.03 %) or due to their own feelings during patient interactions (23.11 %). Lastly, 38.85 % of dentists contemplated leaving the profession.

**Conclusions:**

The impact of the COVID-19 pandemic on dentists was decidedly adverse, manifesting both in personal and professional fields, despite the diverse measures undertaken by these professionals to mitigate its effects.

## 1 Introduction

In December 2019, a new virus was discovered in the Chinese city of Wuhan. Specifically, a new strain of the coronavirus, initially named 2019-nCoV but subsequently renamed SARS-CoV-2. This virus led to the onset of a highly contagious disease, COVID-19. Within a matter of weeks, the virus rapidly disseminated globally, prompting the World Health Organization (WHO) to officially declare it a pandemic by March 2020 [1].

The virus spreads from person to person through direct contact or through aerosols generated by sneezing or coughing [1]. Unvaccinated patients with COVID-19 can range from being asymptomatic to dying. Some symptoms being fever, loss of smell, dry cough, fatigue, expectoration, headache, severe pneumonia, respiratory failure, among others (and depending on the strain). Indeed, the symptoms worsen in people of advanced age or those with pre-existing risk factors [1,2].

Between the months of February and March of the year 2020, the initial cases of people infected with COVID-19 were confirmed and reported in each Latin American country [3]. During that period, effective therapies for treating this virus were not fully established and vaccination campaigns took approximately one year to initiate [4]. Given this scenario, different quarantines and diverse measures restricting people's activities were adopted. The characteristics of these measures and their application times varied among different countries. All of these actions were implemented with the goal of limiting the number of people who became infected and/or died from SARS-CoV-2 [[4], [5], [6]]. As a counterpart, these government actions added damage to the impact that this virus generated on society.

This pandemic has had such disruptive effects that it has generated various problems in different countries; with enormous negative effects on economic, social and health areas [4], particularly during the first year, when the measures to contain the virus's spread were stricter, including substantial restrictions on movement and activities.

Fear, anxiety, anguish, irritability, stress and anger in people are expected feelings in any pandemic [7]. To reduce the impact on health, some governments developed diverse strategies. Different paperwork alert about the negative effects that the COVID-19 pandemic caused on people. For instance, in China, 53.8 % of the population defined the psychological impact of COVID-19 as moderate to severe. In these studies, healthcare personnel were identified as a particularly vulnerable group requiring greater support [4]. The WHO stated that different social groups, delineated by factors such as gender, age, occupation, among others, experience varying degrees of impact from the COVID-19 pandemic. This determines the importance of acknowledging these groups, along with their specific needs and challenges. Recognizing these distinctions makes it possible to offer different interventions for each group [4,8,9].

One of the activities significantly impacted during the first year of the pandemic was dentistry [10,11]. Direct contact with patients as well as aerosols generated during in office procedures are routes for the transmission of SARS-CoV-2 [8,12]. Hence, dental care faced diverse restrictions by official authorities in each country during the initial year of the pandemic. The limitations fluctuated according to the timeframe and geographical region, ranging from the outright prohibition of dental practice to its full operation under strict safety protocols [1]. Initially, routine practices were suspended. Then, they were gradually allowed. However, for most dentists, resuming activities proved to be a gradual and difficult process, as they had to adapt to different evolving regulations. In order to achieve this, they required time for research, acquisition of the different new safety elements and the adaptation of their clinics [10,13].

At the same time, these professionals were also afraid of being infected by patients. Different initial studies determined the fear and anguish suffered by dentists in clinics during the pandemic [10,14,15]. Consolo et al. reported that 85 % of dentists in a province of Italy during the COVID-19 pandemic were concerned about contracting the infection during dental procedures [7]. They also experienced fear (42.4 %), worry (70.2 %), and anxiety (46.4 %) related to attending to patients [7]. Similarly, elevated levels of fear and anxiety were also reported in India.

Conversely, in England 77.2 % of dentists experienced a reduction in income attributable to the pandemic, with 41.1 % reporting a psychological impact [16]. During periods of greatest contagion, a significant proportion of patients canceled their scheduled appointments [7] and a considerable number of dentists reported feeling vulnerable to coronavirus during dental practice [10,17].

Studies of the COVID-19 pandemic, during the vaccination campaign, found that it affected the well-being of dentists, both professionals and students. This phenomenon that has been linked to various variables, including income level, responsibilities for the care of older adults, or even coffee consumption. Additionally, diverse forms of somatizations were reported [18].

The majority of these investigations were conducted through surveys, such as cross-sectional studies [18].

Simultaneously, at times of greatest contagion, a high number of patients canceled their scheduled appointments, with many expressing a sense of vulnerability to coronavirus during dental practice [7,10,17].

For the reasons formerly mentioned, in this work a retrospective study was conducted on the impact the pandemic had on dentists in Latin America and their professional practice during its first year, a period during which vaccination campaigns had not yet started. To achieve this, online surveys with multiple-choice responses were distributed. Subsequently, the obtained results were statistically analyzed. Initially, on a country-by-country basis and finally Latin America as a whole. This approach enabled the identification of specific areas in which dentists were affected and exactly what proportion of them was harmed.

## 2 Material and methods

### 2.1 Sample

The survey was conducted among dental professionals aged 22 or older. All Latin American countries were included: Argentina, Bolivia, Brazil, Chile, Colombia, Costa Rica, Ecuador, El Salvador, Guatemala, Honduras, Mexico, Nicaragua, Panama, Paraguay, Peru, Puerto Rico, the Dominican Republic, Uruguay and Venezuela, except for Cuba, due to local restrictions on communication. The aim was for the survey to reach all specialties of the profession equally, regardless of gender. The sole rectriction for survey participation was being a dentist in one of the countries defined for the study. A total of 1483 dental professionals responded to the survey. The number of dentists across different specialties was not explicitly identified, with the assumption that the proportion of professionals belonging to each specialty in the sample mirrored the actual distribution in the population.

### 2.2 Distribution

The survey was assembled on the Google platform through its “Google Forms” tool, which allowed its virtual distribution over the Internet. Groups of dental professionals from different countries were tracked through Facebook using the Facebook “Group Finder” tool. In total, approximately 80 groups were contacted, and after requesting admission, the survey was subsequently presented. In some countries, they were also sent by email. A pilot test was carried out to assess the distribution, collection of answers and presentation of results. Distribution began on February 22, 2021. The survey remained open for responses for a duration of 40 days. This timeframe was selected as vaccination initiatives were commencing in Latin America, and the intention was to confine responses to the period preceding these vaccination campaigns.

### 2.3 Survey

#### 2.3.1 Survey Structure

The survey was specifically and exclusively targeted at dentists. The survey was composed in two distinct languages: Portuguese for Brazil and Spanish for the remaining Latin American countries. The survey comprised four sections. The presentation section included an informational card containing the informed consent. There was an introduction providing an overview of the study. In addition, contact information for the responsible investigator was provided for direct inquiries about the research. The first section of questions focused on gathering personal data from the dentists, irrespective of the pandemic, including details such as age, years in the profession and other relevant information. This section also included inquiries on local policies regarding pandemic management. The second section of questions centered on the emotional and personal impact on the dentists during the pandemic, regardless of their profession. The third section of questions asked about changes experienced in the professional activity.

The survey consisted of a total of 33 questions. Of these, 31 questions were multiple-choice responses, while the remaining 2 questions required written responses from the participants. The questions permitted the selection of a single answer, except where otherwise specified. Respondents remained anonymous and participation was entirely voluntary. No question was mandatory, except for indicating the country of professional practic (Q4). When referring to the number of surveys collected, it pertains to those surveys where question Q4 minimally answered. The responses to all other questions were consistently considered, regardless of whether other questions were answered or not. Supplementary tables provide a detailed breakdown of the number of completed responses for each question.

The questions are detailed below.

#### 2.3.2 First section of questions

Q1) What is your gender? a) Male. b) Female. *There was no obligation to answer*.

Q2) How old are you? a) between 22 and 30 years old. b) between 31 and 40 years old. c) between 41 and 50 years old. d) between 51 and 60 years old. e) between 61 and 70 years old. f) more than 70 years old.

Q3) How many years of professional practice do you have? a) between 0 and 5. b) between 5 and 15. c) between 15 and 25. d) more than 25 years.

Q4) Where do you practice professionally? A) Argentina. b) Bolivia. c) Brazil. d) Chile. e) Colombia. f) Costa Rica. g) Dominican Republic. h) Ecuador. i) El Salvador. j) Guatemala. k) Honduras. l) Mexico. m) Nicaragua. n) Panama. o) Paraguay. p) Peru. q) Puerto Rico. r) Uruguay. s) Venezuela.

Q5) Do you belong to any risk group for COVID-19? a) Yes. b) No.

Q6) Was individual movement prohibited in your location at any time during the pandemic? a) Yes. b) No.

Q7) Were the necessary personal protective equipment (PPE) and safety protocols for dental work defined locally? a) Yes, but only PPE. b) Yes, but only safety protocols. c) Yes, both. d) No.

Q8) Are official pharmacological therapies applied to treat the coronavirus virus in your locality/country? (do not consider treatments with antisera) a) Yes. b) No.

#### 2.3.3 Second section of questions

Q9) At any time did you feel any of the following feelings when dealing with the COVID-19 pandemic? a) Anxiety. b) Fear. c) Panic. d) Sadness. e) anger f) I did not feel any negative sensation or feeling regarding the pandemic.

Q10) Did you start any psychological or psychiatric treatment because of the pandemic? a) Yes. b) No.

Q11) Did you have to take any medication to feel better mentally during the pandemic? a) Yes. b) No.

Q12) During the pandemic, did you start any new activities to improve your emotional or physical well-being? (for example: doing physical activity at home, starting a hobby at home, fixing unfinished business at home, calling family members or other). a) Yes. b) No.

Q13) If yes, please indicate which:

Q14) At any time during the pandemic, did being close to another person make you nervous or upset you? a) Yes. b) No.

Q15) At any time during the pandemic, did you feel discriminated against for being a dentist? a) Yes. b) No.

#### 2.3.4 Third section of questions

Q16) In which environment do you work? a) Public. b) Private, with my own office. c) Private, without my own office. (this question allowed to select more than one answer).

Q17) Did you stop working in the office at any time during the pandemic? a) Yes, by choice. b) Yes, because the government forbade me. c) Yes, and I still can't return to dental work. d) No. (this question allowed to select more than one answer).

Q18) Did you feel that you were more exposed to infection than other health workers? a) Yes. b) No.

Q19) Do you have all the necessary PPE to work in the office? a) Yes. b) No. c) I still can't return to the profession. (this question allowed to select more than one answer).

Q20) What PPE do you use? a) Surgical mask. b) N95 chinstrap. c) Glasses. d) Mask. e) Scrubs. f) Romper. g) Shoe protector. (this question allowed to select more than one answer).

Q21) Did you have to self-finance the personal protection elements to work in the office? a) Yes. b) No.

Q22) Have you had any difficulties getting the PPEs? a) Yes, because of the costs. b) Yes, because they were scarce. c) I have not yet obtained all the necessary ones. d) No. (this question allowed to select more than one answer).

Q23) Do you feel that the use of PPE or new safety protocols affect the quality of care? a) Yes. b) No.

Q24) How concerned do you think your patients are about contracting COVID-19 during a dental service? a) 1 (not at all and they would go to a consultation without concern). b) 2. c) 3. d) 4. e) 5. f) 6. g) 7 (enough, to the point of not going to the dentist or canceling care already requested).

Q25) Do you feel that the quality of patient care has decreased due to their feelings/sensations regarding the coronavirus? a) Yes. b) No. c) I have no negative feelings about the coronavirus or the pandemic.

Q26) What complications at work did getting the PPEs or following the new safety protocols caused you? a) I was delayed in resuming patient care. b) It still won't let me resume patient care. c) It does not let me see the same number of patients as before. d) I must work more hours than before to attend the same number of patients. e) It is more expensive for me to attend to each patient. f) I cannot perform all dental treatments. g) None. (this question allowed to select more than one answer).

Q27) Do the people who work in the clinic with you have and use both the PPE and the necessary safety protocols? a) Yes. b) No. c) I work alone.

Q28) Rate the complications you had during the pandemic to resume patient care. a) 1 (I have not yet been able to resume patient care). b) 2. c) 3. d) 4. e) 5. f) 6. g) 7 (It was not a problem for me to resume care for patients).

Q29) Did you remotely monitor patients you couldn't see in person during the pandemic? a) No. b) Yes, by phone. c) Yes, by email. d) Yes, by WhatsApp messages. e) Yes, by video call (this question allowed to select more than one answer).

Q30) Were you able to work (non-dental) during the pandemic? a) Yes, in the field of health but independent of the pandemic. b) Yes, in the field of health in actions against the pandemic. c) Yes, I worked but outside the field of health. d) No. (this question allowed to select more than one answer).

Q31) Are you worried about not being able to recover financially after the pandemic? a) Yes. b) No. c) The pandemic did not affect my household income (this question allowed to select more than one answer).

Q32) Did you ever consider giving up dentistry? a) Yes, I considered it. b) Yes, I am still considering it. c) No.

Q33) Do you think that the COVID-19 pandemic will leave any positive element or aspect after it ends? If so, indicate which one.

### 2.4 Statistical analysis

Descriptive statistics was performed. The non-parametric Mann Whitney *U* test (Wilconxon Test) was applied to test differences between groups using the survey results because data was not normally distributed and some samples were small. Consequently, non-parametric data analysis was used.

For each question, the percentage of answers that each option had was established, with its confidence interval (CI) for 95 % using the Wilson/Brown method. This approach was applied to all questions with the exception of two, Q24 and Q28, where the answers were values. In these instances, the CI was calculated using a student's t probability distribution. All calculations were executed using GraphPad Prism 8.0.1 software (GraphPad Software Inc, La Jolla, Calif), specifically utilizing the “Analyze Data/Descriptive Statistics” tool. First, each country was analyzed individually, observing the percentages of responses and their confidence intervals. Subsequently, the results for each question were analyzed for all Latin American countries together, excluding those whose CIs in their responses overlapped with only one or no other country. In essence, when evaluating responses from all of Latin America, countries were excluded when there was statistical significance indicating that their response was different from other countries, even in cases where the overlap occurred with the responses of a single country. This analysis was conducted for each question separately.

### 2.5 Ethics statement

We declare that this study was conducted in accordance with established ethical guidelines and regulations. The participation in the study was anonymous, confidential and voluntary. We do not have mechanisms to link the answers with the names of the individuals who provided them. Informed consent was obtained from all participants.

This study was reviewed and approved by the Comité de Ética y Bioética of the Instituto Universitario Italiano de Rosario by resolution 14/21.

## 3 Results and discussion

### 3.1 Separated country analysis

In the supplementary material, there are tables for each question, providing the total number of responses and the percentage (with 95 % confidence intervals) for each response option, by individual countries. Tables S1, S2 and S3 show the results obtained for questions 1, 2 and 3, respectively. Table S4 shows the results for questions 5 and 6. Tables S5, S6 and S7 show the results obtained for questions 7, 8 and 9, respectively. Table S8 shows the results for questions 10 and 11. Table S9 presents the answers to questions 12 and 14. Tables S10, S11, S12, S13, S14, S15, S16, S17 and S18 present the results for questions 15, 16, 17, 18, 19, 20, 21, 22 and 23, respectively. Table S19 shows the results of questions 24 and 28. Tables S20, S21, S22, S23, S24, S25 and S26 show the results of questions 25, 26, 27, 29, 30, 31 and 32, respectively.

A previously published study evaluated the impact of the pandemic on dentistry in Brazil [6]. The study involved 595 individuals from Brazil, addressing various aspects, including inquiries about emotions. We calculated the CI based on their published data on fear, anxiety, and panic, getting 26.75–19.98 %, 32.33–25.09 % and 3.7–1.28 %; respectively. In our study, 65 Brazilians were surveyed, and the CIs were 33.91–13.73 %, 53.58–29.96 % and 10.86–0.56 %; for fear, anxiety and panic, respectively (Q9, Table S7). Despite the fact that our n was lower than theirs, the CIs overlapped, so it cannot be affirmed with statistical significance that the values obtained are different. Another study conducted in Brazil also researched about the risk factors, just like we did [12]. They surveyed 998 dentists. 20.7 % answered affirmatively, a value that is within our CI (37.34–16.28 %) when we asked the same question in Brazil (Q5, Table S5).

**Fig. 1:**
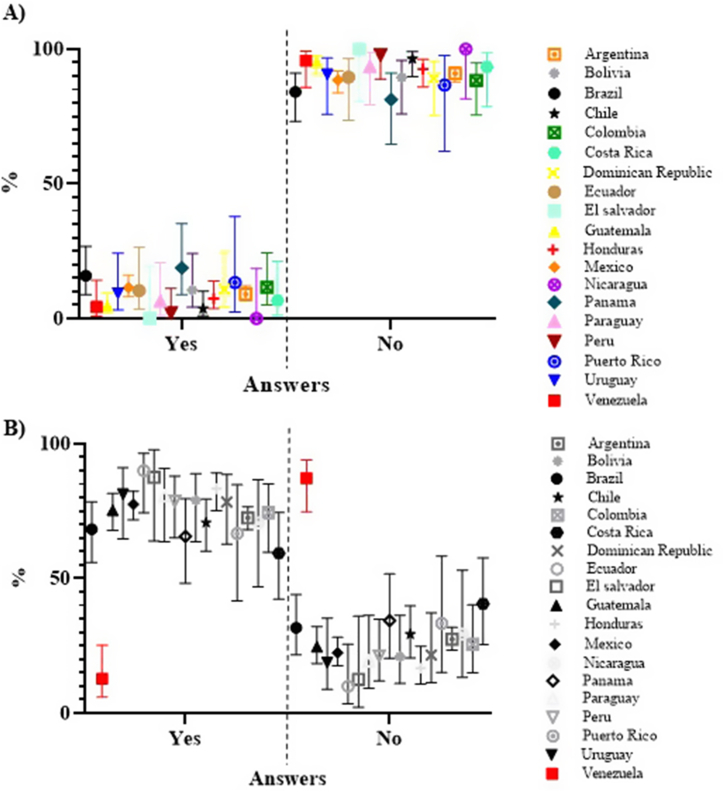
Q10 (A) and Q12 (B) answers of all countries: Percentages of the selected options of the total responses for each country with a 95 % confidence interval.

Looking at the supplementary file tables for each question, it is evident that responses from each country have CIs overlapping with CIs of several other countries, consistently comparing the same response options (see Tables S1–S26). Fig. 1A shows the results of Q10 graphically so as to exemplify what has been explained. But there were 4 questions where this did not occur, that is, where the CIs of the responses of some country did not overlap with any country or only with one:

- Q6 with answers from Uruguay, Nicaragua and Mexico (Table S4). Uruguay and Nicaragua had CI that overlapped only with each other, but with no other country. Mexico only overlapped with a single country.

- Q7 with the answers from Brazil (Table S5).

- Q12 with the responses from Venezuela (Table S9). As an example, the responses from all countries to this question are shown in Fig. 1B.

- Q26 with the responses from Peru (Table S21).

### 3.2 Latin America analysis

In this section, the responses from all of Latin America as a whole are analyzed. In each question, the responses of the 19 countries were considered, except in Q6 (excluding Uruguay, Nicaragua and Mexico), Q7 (excluding Brazil), in Q12 (excluding Venezuela) and in Q26 (excluding Peru). Only those countries whose responses overlap their CIs with more than one country are considered. In these cases, it cannot be affirmed that their answers were different from those of the rest of Latin America (with statistical significance of 95 % confidence). Fig. 1A exemplifies this.

#### 3.2.1 First section

A total of 1483 answered surveys were obtained from the 19 the countries studied. Table 1 shows the number of surveys collected by country of professional practice (Q4). Regarding gender, 68.9 % of respondents identified as female, 31.1 % as male and 0.41 % did provide an answer to this question (Q1).

**Table 1:** Number of surveys answered by country (Q4).

Country	Questionnaires
Argentina	425

Bolivia	38

Brazil	63

Chile	82

Colombia	44

Costa Rica	32

Dominican Republic	37

Ecuador	30

El Salvador	16

Guatemala	150

Honduras	109

México	236

Nicaragua	17

Panamá	32

Paraguay	31

Peru	47

Puerto Rico	15

Uruguay	32

Venezuela	47

**Fig. 2:**
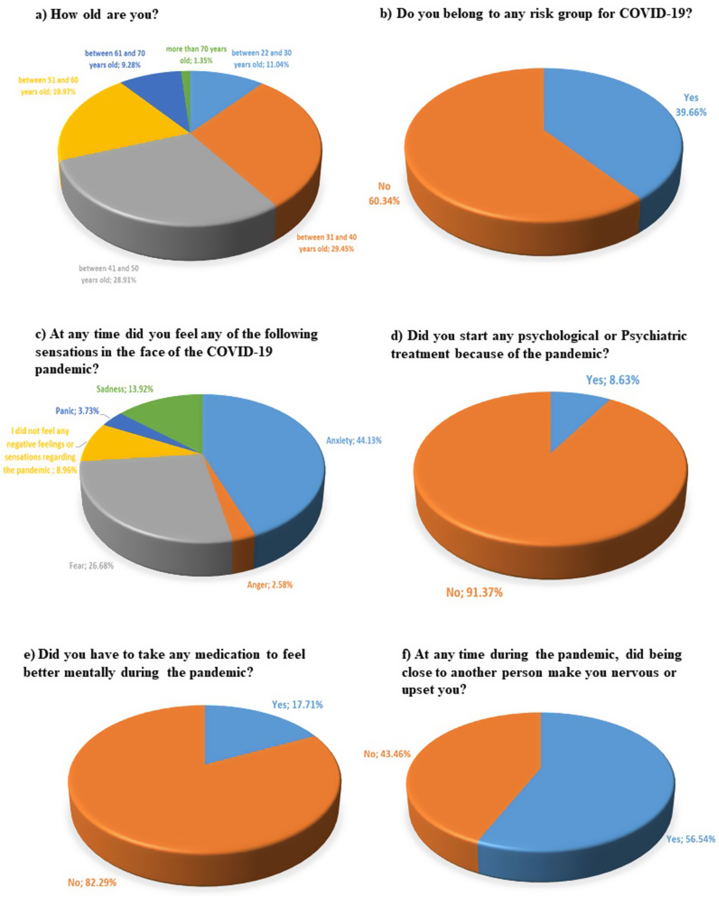
The percentages of the results for all of Latin America: a) Age range of the dentists surveyed (Q2). b) Belonging to a risk group for COVID-19 (Q5) (“Yes”: CI: 42.18–37.19 %, “No”: CI: 62.81–57.82 %). c) Feelings suffered (Q9) (“anxiety” CI: 46.68–41.61 %, “anger” CI: 3.52–1.89 %, “fear” CI: 28.99–24.48 %, “I did not feel any sensation or negative feeling regarding the pandemic” CI: 10.53–7.61 %, “panic”: 4.83–2.88 % and “sadness” CI: 15.78–12.24 %); d) Start of some psychological/psychiatric treatment (Q10) (“Yes” CI: 10.17–7.29 %, “No” CI: 92.70–89.83 %); e) Did you take medication? (Q11) (“Yes” CI: 19.76–15.84 %, “No” CI: 84.16–80.24 %); f) Did being close to another person make you nervous or upset you? (Q14) (“Yes” CI: 59.05–54 %, “No” CI: 46–40.95 %).

Fig. 2 a and 2. b show the results for all of Latin America for Q2 and Q5. Table 2 presents the results for all of Latin America for Q3, Q6 (except for Uruguay, Nicaragua and Mexico), Q7 (except for Brazil) and Q8.

**Table 2:** Percentages of the selected options of the total responses from all of Latin America for Q3, Q6 (except for Uruguay, Nicaragua and Mexico), Q7 (except for Brazil), Q8, Q12 (except for Venezuela), Q13 and Q15. It was calculated with a 95 % confidence interval, defined with an Upper Limit (U.L.) and a Lower Limit (L.L.).

		Mean (%)	U.L. (%)	L.L. (%)
Work experience-Q3	between 0 and 5	13.27	15.1	11.63

	between 5 and 15	27.96	30.31	25.73

between 15 and 25	28.64	31	26.39

more than 25	30.13	32.52	27.84

Circulation prohibited?-Q6	Yes	94.38	95.55	92.93

No	5.62	7.07	4.45

PPE and safety protocols defined-Q7	No	11.88	13.66	10.3

	Yes, both	65	67.44	62.49

Yes, but only PPE	10.82	12.54	9.31

Yes, but only security protocols	12.3	14.11	10.69

Defined protocols?-Q8	Yes	50.32	52.93	47.71

No	49.68	52.29	47.07

New activity? - Q12	Yes	75.14	77.31	72.83

No	24.86	27.17	22.69

Which activity - Q13	Social	18.1	20.63	15.81

	labor	5.11	6.68	3.9

	physical	34.05	37.08	31.15

playful	15.64	18.05	13.5

religious	0.61	1.33	0.28

home	26.48	29.34	23.81

Felt discrimination? - Q15	Yes	35.39	37.87	32.99
	No	64.61	67.01	62.13

It is evident that nearly half of the people who answered the survey are between 31 and 50 years old (Fig. 2a). Observing the overlap of the CIs of the years of professional practice (Q3, Table 2), it can be affirmed that the respondents were evenly distributed according to their work experience, except for those who had an experience of less than 5 years.

A 94.38 % of Latin American dentists (except for Peru) stated that general circulation was prohibited at some point within the first year of the pandemic (Table 2; Q6). Observing the results of Q7 (Table 2), it is only 11.88 % of the respondents affirmed that neither PPE nor safety protocols were defined in their location. Unfortunately, only 65 % of respondents indicated that both PPEs and safety protocols were established in their workplaces. Consequently, it can be asserted that the prohibition of circulation was a measure more frequently adopted than the safety protocols and PPEs. Finally, observing the global results of Q8 (Table 2), it is evident that there was no statistically significant difference in the percentages between the dentists affirming the existence of official pharmacological therapies (50.32 %) and those denying it (49.68 %).

While last questions in the first section are about local regulations, which would be assumed to be the same for each region (Q6, Q7 and Q8), the responses were not the same among dentists in each country (tables S4, S5 and S6). This is attributed to 2 reasons. The first one is that, despite the general regulations of each country, different regions or specific cities within them could have adopted different regulations. The second reason is that dentists could have been wrong in their responses about the inquired aspects. This study was not really interested in how the local situations were, but rather on how the dentists experienced them or how they thought they were. Indeed, this study operated under the assumption that the dentist's personal experiences were what really influenced how they experienced the pandemic.

#### 3.2.2 Second section

Q9 results for all of Latin America are shown in Fig. 2 c. It can be seen that “anxiety” was the option chosen by most professionals (44.13 %), followed by “fear” (26.68 %). Fig. 2 d. Shows the results of Q10. Only 8.63 % required a mental health professional to help them cope with the pandemic. Fig. 2 e shows the results of Q11, indicating that 17.71 % of dentists had to take psychotropic drugs to improve their emotional state during the pandemic. Considering the responses of Q10 and Q11 and that psychotropic drugs must be prescribed by an independent doctor to dentists, approximately half of the professionals took self-medicated drugs.

Table 2 shows the percentages of responses for all of Latin America for questions Q12 (except for Venezuela), Q13 and Q15. It is evident that 3 out of 4 dentists (75.14 %) started some new activity to improve their physical and/or mental well-being (Q12). In question Q13, they were asked to state which activity they had taken up. The responses were varied and were grouped into 6 categories: a) social: activities in which the objective was to get closer to another person (friend or relative), for example: calling their loved ones more frequently, making videoconferences, spending more time with their children, among others; b) labor: activities to generate money as well as to practice or improve their professional practice, for example: teaching classes, taking courses, selling products or others; c) physical: any practice of physical activity; d) entertainment: solitary activity with the exclusive purpose of entertaining oneself, like a hobby; e) religious: exercise directed by a religious or spiritual belief, for example, meditating or praying more frequently; f) home: activity aimed at improving the private home of the respondents. There were 988 responses in Q13. Its distribution into the groups is shown in Table 2. It can be seen that physical activity was the most common answer (34.05 %). The two least frequent activities were work (5.11 %) and religious (0.61 %).

Fig. 2 f shows that more than half of the dentists (56.54 %) expressed discomfort when someone physically approached them during the first year of the pandemic (Q14). Additionally, approximately 1 out of 3 respondents (35.39 %) felt discriminated against at some point due to their profession as dentists (Q15, Table 2).

This section asked about feelings and emotions, but did not seek to make a quantitative or in-depth evaluation of psycho-emotional aspects. The purpose was solely to assess the impact of the pandemic on dentists across various aspects, similar to other studies [6,13,15,18].

#### 3.2.3 Third section

**Fig. 3:**
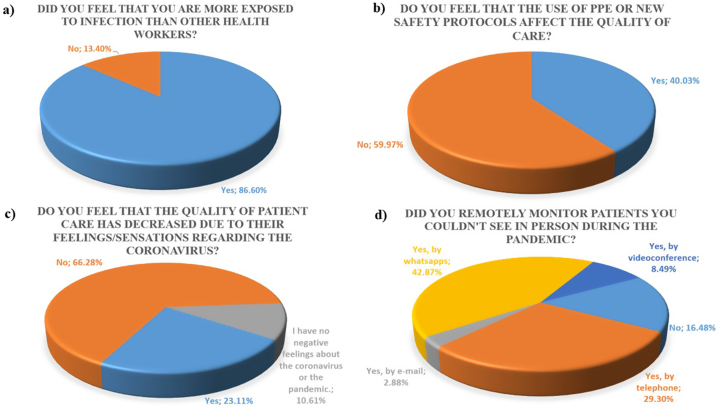
Percentage of responses from all of Latin America: a) Feeling more exposed than other health professionals (Q18) (“Yes” CI: 88.25–84.76 %, “No” CI: 15.24–11.76 %); b) PPEs or new protocols affect the quality of care (Q23) (“Yes” CI: 42.56–37.55 %, “No” CI: 62.45–57.44 %); c) The quality of care for patients decreased due to their feelings (Q25) (“Yes” CI: 25.34–21.03 %, “No” CI: 68.65–63.83 % and “I have no negative feelings about the coronavirus or the pandemic” CI: 12.28–9.13 %) d) Contact with the patient (29) (“No” CI: 18.18–14.91 %, “Yes, by phone” CI: 31.32–27.31 %, “Yes, by email” CI: 3.72–2.23 %, “Yes, by WhatsApp messages” CI: 45.06–40.71 % and “Yes, by video call” CI: 9.80–7.34 %)).

Table 3 shows the percentages of responses for all of Latin America for questions Q16, Q17, Q19, Q20, Q21, Q22, Q26, Q27, Q30, Q31, Q32, and Q33. Fig. 3 shows the results of questions Q18, Q23, Q25 and Q29.

**Table 3:** Percentages of the selected options of the total responses from all of Latin America for Q16, Q17, Q19, Q20, Q21, Q22, Q26 (except Peru), Q27, Q30, Q31, Q32 and Q33. It was calculated with a 95 % confidence interval, defined with an upper limit (U.L.) and a Lower Limit (L.L.).

		Mean (%)	U. L. (%)	L.L. (%)
Where did you work?-Q16	Public	19.3	21.24	17.5

	Private with my own office	62.86	65.11	60.54

Private without my own office	17.84	19.73	16.1

Did you stop working?-Q17	Yes, by choice	43.16	45.54	40.81

	Yes, because the gov. forbade me.	36.15	38.47	33.89

Yes, and I still can't return to dental work	5.23	6.4	4.27

No	15.46	17.26	13.81

Do you have all the PPEs?-Q19	Yes	88.27	89.81	86.53

	No	7.28	8.72	6.07

I still can't return to the profession	4.45	5.62	3.51

What PPEs do you use?-Q20	Surgical mask	13.35	14.16	12.58

	Chinstrap N95	16.32	17.19	15.48

	Glasses	15.93	16.79	15.1

	Mask	18.57	19.49	17.69

Scrubs	16.44	17.32	15.6

Romper	9.1	9.79	8.46

Shoe protector	10.28	11.01	9.6

Self-finance-Q21	Yes	92.05	93.32	90.55

No	7.95	9.45	6.68

Difficulties to get PPEs-Q22	Yes, because of the costs	40.53	42.79	38.31

	Yes, because they were out of stock	37.77	40	35.59

I still haven't gotten all the necessary ones	2.6	3.43	1.96

No	19.1	20.96	17.37

Difficulties to work-Q26	I was delayed in resuming patient care.	10.11	11.27	9.05

	It still won't let me resume patient care.	2.02	2.61	1.56

	It does not let me see the same number of patients as before.	29.4	31.11	27.74

	I must work more hours than before to attend the same number of patients	14.79	16.15	13.53

It is more expensive for me to attend to each patient.	32.59	34.34	30.88

I cannot perform all dental treatments.	7.62	8.66	6.7

None	3.48	4.22	2.86

Do others use PPE and protocols?-Q27	Yes	70	72.29	67.61

	No	5.44	6.72	4.39

I work alone	24.56	26.82	22.43

Non-dental work-Q30	Yes, in the health field but independent of the pandemic.	13.13	14.94	11.52

	Yes, in the health field in actions against the pandemic.	8.6	10.13	7.28

Yes, I worked but outside the health field.	16.8	18.78	14.99

No	61.47	63.9	58.98

Economic concern?- Q31	Yes	63.15	65.56	60.66

	No	23.47	25.69	21.39

The pandemic did not affect my household income.	13.38	15.21	11.75

Drop out of destistry-Q32	Yes, I considered it	24.29	26.57	22.15

	Yes, I am still considering it.	14.56	16.47	12.84

No	61.15	63.62	58.61

Positive aspects the pandemic will leave?-Q33	Biosafety	47.84	50.95	44.75
	Quality of care	3.02	4.27	2.12

	Training	4.12	5.54	3.05

	Quality of life	20.4	23.02	18.01

Family	3.62	4.97	2.62

Reevaluate the activity	9.65	11.64	7.97

No	11.36	13.48	9.53

Among the surveyed dentists, 62.86 % of responses indicated that they work in their private practice, while only 19.3 % indicated that they work in the public sphere (Q16, Table 3). Only 15.46 % of the responses in Q17 indicated that they never stopped working in the office during the first year of the pandemic (Table 3). An 86.6 % of the professionals expressed feeling more exposed to infection than other health workers (Q18, Fig. 3a).

In Q19, 7.28 % of the answers indicated that after a year of pandemic, they could not get all the necessary PPE to work. There was no statistically significant difference regarding the problems that made it difficult to obtain PPEs: costs (40.53 %) and scarcity of materials (37.77 %); but there were 19.05 % of responses indicating that they had no problems obtaining them (Q22, Table 3). 92.05 % of the dentists had to finance the PPEs themselves (Table 3, Q21). Observing the answers to Q20 in Table 3 and it can be highlighted that the most frequently used safety elements were the N95 chinstrap, mask, goggles and scrubs. Finally, 40.03 % of the dentists reported that the new PPEs and safety protocols affect the quality of care (Q23, Fig. 3b).

When dentists were asked to rate the level of concern they thought patients had about attending a dental appointment (Q24), the average value obtained was 4.4 (CI: 4.48–4.31). It should be noted that the extreme values had opposite meanings and the mean value is 4.

The use of new PPEs or safety protocols meant that dentists could not see the same number of patients as before (29.4 %) or found it more expensive to do so (32.59 %). These were the most frequently reported drawbacks. Only 3.48 % of the dentists stated that they had no problem (Q26, Table 3). Fortunately, only 5.44 % of the professionals stated that the people who worked in the clinic with them did not use PPEs or safety protocols (Q27, Table 3).

Fig. 3 c shows the results of question Q25. A 23.11 % of the dentists stated that their feelings or sensations towards the coronavirus did affect the quality of patient care.

When asked if they had followed up patients remotely during the pandemic (Q29, Fig. 3d), 16.48 % of the responses were negative. A 42.87 % indicated that they did, through WhatsApp. When asked if they had worked outside of the dentistry field during the pandemic (Q30), 61.47 % of the responses were negative. Among the affirmative answers, the most frequent one was that they had worked outside the health field, 16.8 % (Table 3).

When respondents were asked to rate the difficulties encountered in resuming care for patients, the average value obtained was 4.66 (CI: 4.74–4.58). The mean value of the scale is 4.

When asked if they were concerned about recovering financially after the pandemic (Q31), 63.15 % of the answers were affirmative (Table 3). Unfortunately, 38.85 % of dentists considered leaving dentistry after the first year of the pandemic (combined percentage, Q32, Table 3).

The last question, Q33, was open-ended. It sought to understand whether the dentist believed that the pandemic had positive consequences and if so, inquired about which ones. Only 11.36 % answered that the pandemic did not have any positive consequence. Within the affirmative answers, the positive aspects described were diverse. As with Q14, they were grouped to analyze them: a) Biosafety: they went from awareness of the various infection risks to changes in practices in the office for a safer exercise, which they assume will continue after the pandemic; b) Quality of care: the process allowed them to reorganize the office, improving its quality; c) Training: generally online, it includes all the courses that dentists were allowed to take to improve their profession; d) Quality of life: includes not just reflecting on life but also involves activities or elements that were acquired during the pandemic to improve quality and that will be maintained after the pandemic ends; e) Family: improvements that favor relations with the family group; f) Review the activity: changes that were made at work but referred to internal management of the office, considerations regarding social works and other aspects that are not directly related to patient treatment. Approximately half of the dentists (47.84 %) who indicated that the pandemic would impact positively referred to biosafety considerations (Table 3).

### 3.3 General discussion

The survey had an internal control to assess the degree of coherence of the respondents. In Q9 and Q25, questions were asked about different aspects, but among the answers to each question there was an option that referred to the same thing: “I did not feel any negative sensation or feeling regarding the pandemic” of Q9 and “I do not have negative feelings about the coronavirus or the pandemic” of Q25. These questions only allowed a single answer to be selected. The CI of the first one was: 10.53–7.61 % and that of the second one was 12.28–9.13 %. Since their CIs overlap, there is no statistical significance to say that their response rates were different, which was expected.

The responses from all of Latin America for each question are presented with their corresponding confidence intervals. Although there may be individual characteristics of each country when looking at the main values of the responses, when one considers the 95 % confidence intervals, it could not be stated with statistical significance that the responses were different between the countries, except in only 4 out of 33 questions. Individual country data is available in supplementary tables. In theses, there are three countries (El Salvador, Nicaragua and Puerto Rico) where low ns occurred. In such cases, the overall results for all of Latin America can be used as particular descriptors for these countries (except for Nicaragua with Q6), given that, with few exceptions, we do not find statistically significant differences between the different countries.

This contrasts with the fact that the socioeconomic and cultural realities of Latin American countries diverge significantly. This situation might be attributed to the fact that the survey was addressed to a very limited sector of society, a specific group of university professionals with greater similarities between the countries. The similarity among counties is also favored by the international connection of our times. The WHO makes recommendations that are immediately received anywhere in the world [8]. Similarly, measures specific to one country can spread with the same speed to the others.

Concurrently, dentists typically operate outside a dependency employment relationship, and as such, they often lack defined limits imposed by work structures. However, this independence comes with fewer elements of labor protection. Therefore, in a crisis like the pandemic, they are notably vulnerable [19]. For instance: 92.05 % had to self-finance the personal protective equipment (PPE). This situation is similar in all Latin American countries and could contribute to the results of the surveys did not differ with statistical significance among the different countries.

As mentioned earlier, our findings align with those reported by other researchers in Brazil [6,12]. When examining similar survey results outside of Latin America, similar results can be observed regarding the level of self-sufficiency that dentists had during the pandemic (Nepal) [20], financial concerns (Italy), desires to leave the profession (Italy) [21], or worsening of different aspects in patient care (U.S.A.) (22).

The most remarkable contributions of our study were: a) the absence of statistical differences among the countries of Latin America, b) 8.63 % of surveyed individuals sought the assistance of psychologist or psychiatrist, c) 17.71 % initiated the consumption of some psychoactive drug during the first year, d) 40.03 % stated that the quality of care decreased due to the new safety measures and e) 38.85 % of dentists considered giving up the profession in the first year.

Regarding potential biases of the results, the foremost and critical consideration is that the findings are derived solely from individuals who voluntarily chose to participate in the study. Another bias lies in the nature of self-reported responses, mainly in the self-assessment of the feelings suffered by the pandemic, with a focus on “anxiety”. This sentiment is conceptually broad and may not be as clearly understood compared to others such fear, panic, sadness or anger. Finally, another significant bias is that this survey was exclusively answered by individuals who participate in Facebook professional groups.

Individuals who underwent quarantine during the pandemic were more prone to suffer emotional disturbances due to the extensive preventive, isolation and protection measures implemented. Dentists used personal protective equipment to avoid infections. However, this equipment led to discomfort, contributing to heightened work-related stress. In Latin America, where the health systems of certain countries face limitations, the damage was magnified [19].

In situations with diverse and profound negative impacts, it is relevant to identify all influencing variables to formulate various interventions across different stages of the pandemic. Certain studies have determined deficiencies in the policies developed by the authorities [4]. Addressing the impact of the pandemic on health considering all factors is paramount to prevent harm other than physical damage. Therefore, studies of this nature are essential for a comprehensive understanding of the pandemic [4,9]. Our research provided insights into specific aspects in which dentists were adversely affected and precisely quantified the proportion of the population impacted.

## 4 Conclusions

Many pathogens have different ways of affecting health, some even more aggressively than SARCOV-2 [20,21]. However, in this specific pandemic the impact on society was enhanced by the measures of local governments which, trying to reduce damage in the area of physical health, implemented measures that increased damage in other areas, such as economic, psychological, social, labor, among others. The objective of this study was to evaluate how the pandemic affected dentists in Latin America, beyond their health, during the first year, when there were no vaccination campaigns.

1,483 surveys were collected and approximately half of them were answered by professionals between the ages of 31 and 50; and in general, the distribution of job seniority of the respondents was uniform.

Governments adopted different measures to reduce contagion in the population. A fairly common one was to prohibit circulation (94.38 % of dentists suffered from it). All measures, directly or indirectly, affected the population. For instance, over half of the dentists (56.54 %) stated that they suffered discomfort when physically approached by another person. Approximately one out of three respondents acknowledged feeling discriminated against due to their profession at some point. An 86.6 % of the professionals expressed feeling more exposed to becoming infected than other workers in the health area. To mitigate the impact, many individuals initiated various activities. In this survey, two out of three dentists reported adopting new activities to alleviate the discomfort of the pandemic. Even so, 8.63 % had receive help by a psychologist or psychiatrist and 17.71 % commenced the consumption of psychoactive drug.

Regarding the practice of the profession, dentistry suffered different kinds of restrictions. In several countries this practice was prohibited at first. Only 15.46 % of the dentists indicated that they never stopped working in the office during the first year of the pandemic. Fortunately, only 16.48 % of the professionals did not control their patients remotely.

Practices were eventually permitted with the implementation of PPE and safety protocols. However, even after a year, 7.28 % of professionals indicated that they had not yet obtained all the necessary PPE. Dentists requiring these supplies for their practices did not receive financial assistance; instead, 92.05 % reported having to cover the expenses personally.

When asked specifically about quality, 40.03 % indicated a decline due to the implementation of the new safety measures. Concurrently, quality was also affected by the dentists’ own feelings in 23.11 % of the respondents.

Another aspect that deeply affected dentists was their home economics. This can be interpreted since almost 2 out of 3 stated that they were concerned about being able to recover in this sense.

Our study is subject to certain biases: the results are derived solely from individuals who voluntarily chose to participate in the study, the responses are self-reported and the survey was exclusively answered by individuals who are participants in professional groups on Facebook.

The objective of this work was to quantify what proportion of dentists had been negatively affected by various areas during the first year of the pandemic, prior to the implementation of vaccines. However, the question that probably best summarizes the magnitude of the damage to their profession was Q32. More than a third of dentists who answered this question considered leaving the profession after experiencing the first year.

Government and administration institutions must take general and particular measures to reduce the impact when dealing with health crisis. Dentists are a specific group of the population: they are health professionals who were especially vulnerable in the COVid-19 pandemic due to the characteristics of their practices. In order to protect them properly, more specific measures had to be taken. For instance, there is a need for the development of diverse activities accessible to the general population for home-based engagement to mitigate the negative effects of confinement. Collaborative efforts could be established with psychology associations to offer virtual psychological assistance tailored to the specific needs of the profession. Professional dental associations could provide recommendations for conducting remote check-ups on patients, considering that only 16.48 % reported not doing so in practice. Furthermore, authorities should facilitate access for dentists to acquire necessary safety equipment.

This study provided insights into the specific areas where dentists were affected and precisely determined the proportion of the population impacted. Future psychological studies should further explore and measure the level of reported emotions. Our research group plans to conduct a cross-response study with the data collected from this same survey.

## Data availability statement

The data associated to this study has not been deposited into a publicly accessible repository. However, upon request, we will make all data available.

## Ethics statement

We declare that this study was conducted in accordance with established ethical guidelines and regulations. Informed consent was obtained from the participants.

This research project was approved by the Comité de Ética y Bioética of the Instituto Universitario Italiano de Rosario by resolution 14/21.

## Funding

This research did not receive any specific grant from funding agencies in the public, commercial, or not-for-profit sectors.

## CRediT authorship contribution statement

**Juan Gabriel Costa:** Writing – review & editing, Writing – original draft, Visualization, Validation, Supervision, Software, Resources, Project administration, Methodology, Investigation, Formal analysis, Data curation, Conceptualization. **Ana Beatriz Gaudio:** Investigation, Data curation, Conceptualization. **Nicolás Gomez Giorgi:** Investigation, Data curation, Conceptualization. **Camila Hanow:** Investigation, Data curation, Conceptualization.

## Declaration of competing interest

The authors declare that they have no known competing financial interests or personal relationships that could have appeared to influence the work reported in this paper.
